# Effect of Immunocastration and Diet on Growth Performance, Serum Metabolites and Sex Hormones, Reproductive Organ Development and Carcass Quality of Heavy Gilts

**DOI:** 10.3390/ani11071900

**Published:** 2021-06-25

**Authors:** Leticia Pérez-Ciria, Francisco Javier Miana-Mena, María Victoria Falceto, Olga Mitjana, Maria Angeles Latorre

**Affiliations:** 1 Departamento de Producción Animal y Ciencia de los Alimentos, Instituto Agroalimentario de Aragón-IA2, Universidad de Zaragoza-CITA, 50013 Zaragoza, Spain; leticiapcgm@gmail.com; 2 Departamento de Farmacología y Fisiología, Instituto Agroalimentario de Aragón-IA2, Universidad de Zaragoza-CITA, 50013 Zaragoza, Spain; jmiana@unizar.es; 3 Departamento de Patología Animal, Instituto Agroalimentario de Aragón-IA2, Universidad de Zaragoza-CITA, 50013 Zaragoza, Spain; vfalceto@unizar.es (M.V.F.); omitjana@unizar.es (O.M.)

**Keywords:** immunocastration, dietary energy, dietary protein, growth performance, sex hormones, reproductive organs, carcass quality, gilts

## Abstract

**Simple Summary:**

Currently, a considerable proportion of the carcasses intended for the production of Teruel dry-cured ham are declared unsuitable for this purpose, mainly due to their lack of fat. This problem is detected especially in females, because males are castrated to avoid boar taint, and castration increases fat deposition. Immunocastration (immunization against gonadotrophin releasing factor) could resolve this issue, as that collateral effect has been reported in the literature. Increasing energy or reducing protein and amino acids in the diet could also result in greater fatness. Additionally, immunocastrated gilts could have different feeding pattern to entire females, and thus it is interesting to study feeding plans. Therefore, a study was conducted to evaluate the influence of immunocastration and provided diet on growth performance, serum metabolites and sex hormones, reproductive organ development, and carcass quality. In this trial, it was concluded that immunocastration is a positive strategy to apply in gilts intended for Teruel dry-cured ham production, because it increases growth rate and fatness. On the other hand, irrespective of immunocastration, a rise in dietary energy or a drop in dietary crude protein and amino acids in gilts from 76 to 134 kg could also be beneficial strategies for pig farmers.

**Abstract:**

It is desirable to increase fatness in gilts destined for Teruel dry-cured ham production. A total of 192 Duroc × (Landrace × Large White) gilts of 40.3 ± 4.80 kg body weight (BW) were used to assess the impact of immunocastration and feeding on growth performance, serum metabolites and sex hormones, reproductive organ development, and carcass quality. Six treatments were arranged factorially (2 × 3) with two types of gilt (entire gilts (EG) vs. immunocastrated gilts (IG)) and three experimental diets (control vs. high energy vs. low crude protein and amino acids) provided from 76 to 134 kg BW (*n* = 4 per treatment, being the replicate the pen with eight pigs). Immunocastration was carried out at 58 and 77 kg BW. The IG grew faster and showed lighter reproductive tracts and greater fatness than EG. The experimental feeds had limited effect on carcass quality, but the high-energy diet improved gain-to-feed ratio and the low-protein and -amino-acids diet did not impair growth performance. In conclusion, immunocastration was a better strategy than the tested diets to increase the fatness of gilts intended for Teruel dry-cured ham, although increasing energy or decreasing crude protein and amino acid levels in the diet could be beneficial strategies for pig farmers.

## 1. Introduction

“Teruel ham” is a Spanish protected designation of origin (PDO) dry-cured ham with national relevance and certain international recognition. It is based on white-crossbreed pigs (not autochthonous) slaughtered at a heavy body weight (BW) (around 135 kg). To guarantee the quality and homogeneity of the end product, the Consortium of that PDO requires a fat depth over the gluteus medius muscle (GM) > 16 mm [1], which is necessary to optimize the dry-curing process [2]. The Teruel ham industry complains about the difficulties in achieving that fat thickness, with this problem being detected especially in females [3], because males are surgically castrated to avoid boar taint, and castration increases subcutaneous fat content [4]. One strategy to try to resolve this issue could be the castration of gilts, which would be expected to produce a similar effect to that in males. However, surgical castration of female pigs reared indoors is banned in the European Union [5]. Therefore, immunocastration might be an alternative. It consists of the application of several vaccines in which the active substance is a gonadotrophin-releasing factor (GnRF) analogue–protein conjugate. This analogue stimulates the gilt immune system to produce anti-GnRF antibodies that neutralize the pig’s GnRF, blocking the production of follicle-stimulating hormone and luteinizing hormone. Therefore, estrus is temporarily suppressed [6]. Additionally, immunocastrated gilts (IG) could have different feeding pattern to entire females (EG); thus, it is reasonable to study feeding plans in this type of animals, which might provide positive or even additional effects to immunocastration on animal fattening. In this sense, the increase of dietary energy level and the reduction of crude protein (CP) and amino acids (AA) contents could be interesting, because these strategies seem to be successful in EG [7,8]. Therefore, the objective of this trial was to evaluate the impact of immunocastration and diet on growth performance, serum metabolites and sex hormones, reproductive organ development, and carcass quality of gilts intended for Teruel dry-cured ham production.

## 2. Materials and Methods

### 2.1. Animal Husbandry and Experimental Design

All the experimental procedures used in this experiment followed the ethical committee requirements of the University of Zaragoza (ref. PI29/18). Animals were cared for and managed according to the Spanish Policy for Animal Protection [9]. A total of 192 Duroc × (Landrace × Large White) gilts with 40.3 ± 4.80 kg BW (84 ± 3 days of age) were used. On the arrival at the farm (Foz-Calanda, Teruel, Spain), pigs were individually identified and weighed, and housed in groups of eight in pens of 9 m^2^ with 50% slatted floor, equipped with one drinking cup and one grow feeder. Animals were allotted to blocks of increasing BW and each block contained all treatments.

Half of the gilts were immunocastrated by injection of two doses of Vacsincel^®^ (Zoetis Spain S.L., Alcobendas, Madrid, Spain), with the first injection at 58.1 ± 6.39 kg of BW (102 ± 3 days of age) and the second one at 77.0 ± 8.12 kg of BW (122 ± 3 days of age), according to the conclusions obtained in a previous study [10]. The other half of the gilts remained intact throughout the trial. Three experimental diets were offered to both IG and EG during the grower and finisher periods (Table S1 and Table 1): (i) a control diet with a similar nutritional profile to the recommendations of FEDNA [11]; (ii) a diet with a greater energy content than the control diet, but with similar CP and AA percentages; and (iii) a diet with lower CP and AA contents than the control diet, but with the same energy level. In all diets, ideal protein content was maintained [11] and the change between the grower and the finisher feeds was carried out on a fixed day. The grower diets were supplied from 122 to 149 ± 3 days of age (76–102 kg BW) and the finisher diets were given from 150 ± 3 days of age to the day before slaughter (102–134 kg BW). Therefore, there were six experimental treatments; two types of gilt (EG vs. IG) × three diets (control vs. high energy vs. low CP and AA).

### 2.2. Feed Sypply and Analyses

Feed in pellet form and water were provided ad libitum throughout the trial. Gross energy of the experimental diets was analyzed using an adiabatic bomb calorimeter (Model 1356, Parr Instrument Company, Moline, IL, USA). Dry matter, ash, and CP were determined following methods 934.01, 942.05, and 2001.11, respectively, of the Association of Official Analytical Collaboration (AOAC) International [12]. Ether extract was analyzed using the Am 5-04 procedure of the American Oil Chemists’ Society [13]. Neutral detergent fiber was determined with an ANKOM^220^ Fiber Analyzer (Ankom Technology, Macedon, NY, USA) as described by Mertens [14]. Starch content was analyzed enzymatically using a commercial kit (Total Starch Assay Kit K-TSTA 07/11, Megazyme, Bray, Wicklow, Ireland). The AA profile was determined in an external laboratory (Ofice S.L., Castellgalí, Barcelona, Spain) using high-performance liquid chromatography–fluorescence.

### 2.3. Growth Performance Measurements

Individual BW was recorded several times: at the beginning of the study, when the first dose of Vacsincel was applied, when the second dose was injected (coinciding with the first day pigs were given the experimental grower diets), the first day of the experimental finisher diets, and the day before slaughter. The records were used to calculate average daily gain (ADG) per pen for each phase and for the overall trial period (from 84 ± 3 days of age to the day before slaughter). Feed consumption per pen was controlled during the grower and the finisher periods to calculate average daily feed intake (ADFI) per pen. Finally, ADG and ADFI per pen were used to calculate gain-to-feed ratio (G:F).

### 2.4. Blood Sampling and Analyses

A blood sample of 10 mL from one pig per pen, chosen at random, was taken at several points: the days on which immunocastration doses were administered (the second one coinciding with the beginning of the grower period), at the end of the grower period, and at the end of the finisher period (coinciding with the day before slaughter). The sampled animals were always the same, and each blood sample was obtained by jugular venipuncture and introduced into a sterile tube with no additives (Vacutainer Brand, Becton Dickinson Vacutainer Systems, Plymouth, Devon, UK). It was conserved at 4 °C until its centrifugation (1600× *g*, 10 min, 4 °C) and serum was then stored at −20 °C. Serum analyses were carried out by an external company (Laboratorios Albéitar, Zaragoza, Spain).

In the case of serum metabolites, the concentrations of albumin, urea, cholesterol, and triglycerides were evaluated at the end of the both the grower and the finisher diets using GernonStar equipment (RAL S.A., Barcelona, Spain). Albumin was analyzed via a colorimetric method (reagent GN 86125); intra-assay coefficient of variation (CV) was 0.50% and inter-assay CV ranged between 0.70 and 0.80% (5.70 and 3.35 g/dL, respectively), depending on the concentration. Urea was determined via a kinetic method (reagent GN 70125); intra-assay CV ranged between 0.77 and 2.79% (120 and 37 mg/dL) and inter-assay CV between 1.65 and 2.65% (126 and 40 mg/dL). Cholesterol and triglycerides were analyzed with an enzymatic–colorimetric method (reagent GN 21100 for cholesterol and GN 90125 for triglycerides). Cholesterol intra-assay CV ranged between 0.76 and 1.22% (185.3 and 99.8 mg/dL) and inter-assay CV between 4.36 and 6.91% (96.3 and 185 mg/dL). Triglycerides intra-assay CV ranged between 0.99 and 1.57% (196 and 128 mg/dL) and inter-assay CV between 3.15 and 7.70% (126 and 201 mg/dL).

In the case of sex hormones, the concentrations of progesterone and estradiol were evaluated on the days of application of the immunization doses and on the day before slaughter using competitive immunoassays with enzyme-labeled chemiluminescent technology (IMMULITE, Siemens Healthineers, Madrid, Spain). Progesterone total CV was 6.5–13.2% over the calibration range of 31.4–1.04 ng/mL, respectively. Estradiol intra-assay CV was 6.3–15% at 480–46 pg/mL, respectively, and inter-assay CV was 6.4–16% over the calibration range of 482–56 pg/mL, respectively.

### 2.5. Slaughtering, Reproductive Organ Collection, and Carcass Measures

The slaughter was planned at a fixed BW (close to 135 kg), and thus pigs were slaughtered at 178, 185, and 199 ± 3 days of age. On the day before slaughter, feed was withdrawn for 5 h and pigs were transported 130 km to a commercial abattoir (Cartesa, Teruel, Spain), where they were kept in lairage for 10 h with full access to water but not to feed. At the slaughterhouse, animals were stunned in CO_2_ atmosphere, exsanguinated, scalded, dehaired, singed, and eviscerated. The genital tracts of a total of 27 gilts (12 EG and 15 IG, chosen at random, which had eaten the same feeding plan (high-energy diet)) were collected in individual plastic bags and conserved at 4 °C until their subsequent evaluation in the laboratory.

For the study of carcass quality, a total of 132 gilts were chosen at random, being 22 from each experimental treatment (type of gilt × diet). After carcasses were split lengthwise, hot carcass weight was individually recorded to calculate carcass yield. Fat depth (skin included) over the GM (at its thinnest point), ham length (from the anterior edge of the pubis symphysis to the hock joint), and ham perimeter (at its widest side) were measured on the left side of each carcass. After refrigeration at 2 °C (approximately 1 m/s air speed and 90% relative humidity) for 5 h, carcasses were processed, and, to fit commercial requirements (round shape), hams and shoulders were trimmed of external fat. The ham and the shoulder from the left side of each carcass were then individually weighed to calculate their yields in the carcass.

### 2.6. Study of Reproductive Organs

The collected genital tracts were dissected and each part was studied separately. Uterine horns, uterine corpus, cervix, and vagina were weighed and their lengths were measured. Vaginal vestibule and vulva were also measured. Each ovary was weighed and its length, width, and depth were measured. The follicles of each ovary were counted according to their size (<2 mm: very small, 2–4 mm: small, 4–6 mm: intermediate, and >6 mm: big follicles) [15].

### 2.7. Statistical Analyses

The Statistical Analysis System, Version 9.4 (SAS Institute Inc., Cary, NC, USA), was used. In the case of growth performance, data were analyzed as a randomized complete block design with a 2 × 3 factorial arrangement of treatments using the GLM procedure, with the pen as the experimental unit (*n* = 4 per treatment). The model included type of gilt (EG or IG) and diet (control, high energy, or low CP and AA) as main effects and the initial BW as the blocking criterion. Interaction (type of gilt × diet) was removed from the final models because it was nonsignificant (*p* > 0.05).

Serum metabolites were analyzed by repeated-measures analysis using the MIXED procedure. The model included type of gilt (EG or IG), diet (control, high energy, or low CP and AA), sampling time (at the end of the grower period or at the end of the finisher period), and their interactions as fixed effects, with the gilt as the experimental unit (*n* = 4 per treatment at each sampling time). Unstructured, compound symmetry, unstructured, and variance components were the covariance structures chosen for albumin, urea, cholesterol, and triglycerides, respectively, since these were some of the models with the smallest Akaike and Bayesian information criteria values.

Progesterone was not statistically analyzed because many values in both types of gilt were below the detection level of the equipment used (0.20 ng/mL); consequently, a descriptive analysis was carried out. Estradiol was analyzed using the MIXED procedure with repeated measures. The model included type of gilt (EG or IG), sampling time (at first dose of immunocastration, at second dose, or the day before slaughter), and their interaction as fixed effects, and gilt within type of gilt as the experimental unit (*n* = 12 per type of gilt at each sampling time). The effect of the diet on sex hormones was not analyzed. Compound symmetry was the covariance structure chosen, since it was the model with the smallest Bayesian information criterion value.

Reproductive organs were analyzed using the GLM procedure. The model included type of gilt (EG or IG) as main effect and the gilt as the experimental unit (*n* = 12 for EG and *n* = 15 for IG). The number of ovarian follicles and the percentage of gilts with follicles in each size category were analyzed using the GENMOD procedure. In the first case, a negative binomial distribution was applied, and in the second one, a binomial distribution was considered. Least square means and 95% confidence intervals were transformed from the log and logit scales, respectively.

Carcass quality was analyzed as a factorial design (2 × 3) using the GLM procedure with the gilt as the experimental unit (*n* = 22 per treatment). The model included type of gilt (EG or IG) and diet (control, high energy, or low CP and AA) as main effects. Interaction (type of gilt × diet) was removed from the final models because it was nonsignificant (*p* > 0.05). Additionally, slaughter weight was included as a covariate in parameters for which it was significant (*p* < 0.05).

Tukey test was used to analyze the differences between least square means for all parameters studied.

Normality of the residuals was checked with Shapiro–Wilk test using the UNIVARIATE procedure. In cases in which normality was not achieved, variables were transformed with $\sqrt{x}$ or Napierian logarithm or $\;x^{3}$ prior to statistical analysis. In these cases, the results are presented as back-transformed least square means with 95% confidence intervals within parentheses. A *p*-value < 0.05 was considered a significant difference and a *p*-value between 0.05 and 0.10 a tendency.

## 3. Results and Discussion

Except for serum triglyceride concentration, the rest of the results are presented as main effects, since no significant interactions were detected.

### 3.1. Growth Performance

Table 2 shows the effect of immunocastration and diet on growth performance of heavy gilts. From the first to the second dose of immunization against GnRF, no effect (*p* = 0.222) was observed in ADG, confirming the findings of a great number of works in this field [6,16,17]. This was expected because the first dose only primes the immune system of the pig [18]. From the second dose of immunocastration to the time of slaughter (coinciding with the overall experimental diet period), IG ate more feed (*p* = 0.006) and grew faster (*p* = 0.002) than EG, with no difference in G:F (*p* = 0.292). This is in agreement with the results of other authors [6,19]. These effects of immunocastration were greater in the finisher phase (from approximately 100 kg BW to the slaughter) by 8% in ADFI (*p* = 0.0005) and by 11% in ADG (*p* = 0.001). In the grower period, the differences were in the same direction but lower; in ADFI it was only a trend (*p* = 0.098) and in ADG it was only numerical (*p* = 0.175). Daza et al. [17] detected higher ADG in IG just after the second injection, and the reason could be that the second dose was applied earlier in their trial. The higher voluntary appetite detected in IG in the current experiment could be explained by a quieter behavior, although this was not evaluated. In male pigs, it has been seen that immunocastration reduces aggressive and sexual behaviors after the second dose [20], which could increase visits to the feeder in group-housed pigs, leading to an increase in feed intake [21]. The lack of effect found in the current trial on feed efficiency was not reported by some other authors. From the second dose to time of slaughter, Bohrer et al. [16] detected that immunocastration improved G:F, and Gómez-Fernández et al. [22] observed the opposite effect. The different responses reported in literature about the impact of immunocastration on growth performance might be attributed to the different genetic used, age, and weight of gilts when the immunization doses were applied and time elapsed between the second dose and the slaughter. For the overall trial period (from 40 kg BW to slaughter), IG showed greater (*p* = 0.0007) ADG than EG, and as a consequence, IG needed 7.4 days less (*p* = 0.005) on the farm to achieve the slaughter weight target, representing a great advantage for pig farmers.

Regarding feeding, no significant differences (*p* > 0.05) between diets were detected in any parameter during the grower period (from approximately 75 to 100 kg BW). The lack of effect of increasing dietary energy while maintaining CP and AA contents agrees with the work of Knowles et al. [23] in similar pigs. Additionally, the lack of influence of decreasing dietary CP and AA levels confirms the results of Pires et al. [24], but disagrees with other authors who detected higher ADFI and worse G:F with restricted diets [23,25] in that range of BWs. These discrepancies could be mainly explained by the different intensities in the decrease in the CP and AA levels tested, being less pronounced in our case. During the finisher period (from approximately 100 to 135 kg BW), gilts fed the diet with high energy level and that with low CP and AA contents grew faster (*p* = 0.002) than those fed the control diet. Pigs that ate the high-energy diet showed lower (*p* = 0.035) ADFI than those that ate the low-CP and -AA diet, with those fed the control diet being in an intermediate position. Thus, gilts fed the high-energy diet presented greater (*p* = 0.005) G:F than those fed the control diet, with those that ate the low-CP and -AA diet being in an intermediate position. With respect to increasing dietary energy level, the G:F result agrees with Suarez-Belloch et al. [7], but the reasons are different. In the case of the latter report [7], the result was due to a lower feed intake with similar daily BW gains, justifying the idea that growing pigs adjust their feed consumption to maintain their voluntary energy intake constant under a wide range of dietary energy concentrations [26]. In the current trial, it was because of a higher ADG with similar ADFI. Our hypothesis is that in pigs above 100 kg BW with great capacity of the digestive tract, and especially under commercial conditions, energy intake is probably below the potential for maximum energy intake and, therefore, an increase in the energy of the diet would not decrease feed intake, thus increasing growth rate, according to the work of De la Llata et al. [27]. Regarding the decrease of dietary protein and AA level during the finisher period, the results obtained were not expected. There is a considerable unanimity in the literature about the lower ADFI when CP and/or AA are restricted, accompanied with worse ADG and feed efficiency [28,29]. However, in the current experiment, the response was a higher ADG, similar to that presented by pigs fed the high-energy diet, and similar ADFI and G:F to control pigs. We do not have an explanation for these results; maybe the restriction in AA levels was very limited.

Finally, evaluating the overall period during which experimental feeds were provided (approximately from 75 to 135 kg BW), only G:F was significantly (*p* = 0.037) different among dietary groups; gilts fed the high-energy diet had greater G:F than those fed the control diet, with animals fed the low-CP and -AA diet in an intermediate position. Therefore, the effects of diet detected in the finisher period were mitigated in the global period because of the lack of impact during the grower phase. These results imply that increasing the dietary energy in gilts destined for Teruel ham from 76 to 134 kg BW could compensate pig farmers, depending on the price of fat sources. Additionally, in this period and in these types of gilts, the application of a diet with low CP and AA contents would reduce feeding cost, because this diet is cheaper, without penalizing growth performance, as well as contributing to reduced nitrogen losses to the environment [30]. When the period of administration of the experimental diets was broader (around 30–115 kg BW), several authors [31,32,33] found no effect of increase dietary energy on ADG and ADFI. Regarding feed efficiency, Marçal et al. [32] obtained similar results as ours, but other authors [31,33] did not observe an impact of increased energy level. In relation to the dietary low CP and AA, when the diet supplementation period was broader (around 20–120 kg BW), several studies [34,35,36] have reported no effect of restricted diets in growth performance, as in the current trial. However, it has to be considered that worse feed conversion ratio and slower growth has been shown by others [37,38]. On the other hand, Schiavon et al. [39] found that pigs fed at low CP and AA levels from 86 to 145 kg BW grew faster and ate more feed than those fed at high CP and AA levels, because many of the pigs restrictively fed the low-CP and -AA diet were forced to consume more feed. These authors [39] suggested that an animal will eat sufficiently to satisfy its genetic requirements for nutrients, even though some factors (diet, climate, disease, or housing) may cause it to either increase or decrease feed intake from its potential.

### 3.2. Serum Metabolites

As can be seen in Table 3, immunocastration had no effect (*p* > 0.05) on concentration of serum metabolites (data of triglycerides not shown). Van den Broeke et al. [19], injecting the second GnRF vaccination at a heavier BW (at 105 kg BW), did observe at slaughter time that gilt immunocastration increased serum urea, and their justification was the higher daily protein intake. In the current trial, IG also showed higher urea content than EG, but the difference was only numerical (nonsignificant).

No influence (*p* > 0.10) of experimental diets was observed on serum albumin, urea, and cholesterol contents. These results are in agreement with previous reports [23,33,40] in which diets with different energy and similar CP and AA contents were compared. On the other hand, when a greater and earlier restriction of CP and AA was practiced, Mule et al. [41], Ruusunen et al. [42], and Suárez-Belloch et al. [43] detected lower albumin level, and Chiba et al. [44], Fabian et al. [45], and Kerr et al. [46] observed lower serum urea concentration. The authors explained these results; the effect on albumin was because the limitations in the availability of AA first appear in the synthesis of exported proteins [42], and the effect on urea was due to a lower nitrogen intake [39], implying that pigs fed low levels of CP and AA use nitrogen more efficiently for growth [47]. Therefore, the results of the current trial about serum albumin and urea confirm the previous idea of a limited CP and AA restriction. Suárez-Belloch et al. [43] found an increase in cholesterol concentration associated with limited CP and AA in diets, indicating a possible hypercholesterolemic effect in those animals [41]. Additionally, it is worth noting that as pigs grew older, they showed greater concentrations of albumin, urea, and cholesterol (*p* = 0.0001, *p* = 0.011, and *p* = 0.012, respectively).

**Figure 1:**
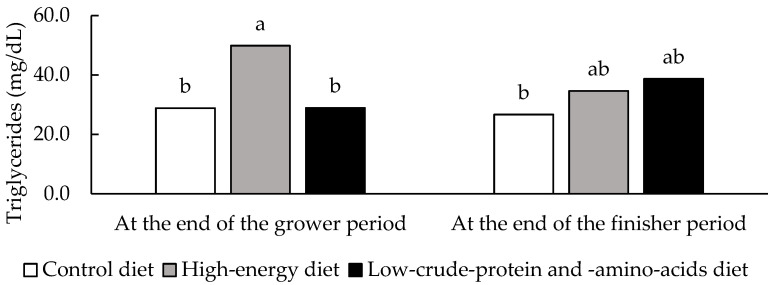
Significant interaction (*p* = 0.031) between diet and sampling time on serum triglyceride concentration of heavy gilts. The average body weight of gilts at the end of the grower period was 102 kg and at the end of the finisher period was 134 kg. Statistical evaluation was carried out with data after their transformation. Data are presented as back-transformed least square means. Different letters (a,b) denote significant differences between treatments (*n* = 8) (*p* < 0.05).

A significant interaction (*p* = 0.031) between diet and sampling time on serum triglyceride concentration is shown in Figure 1. At the end of the grower period, gilts fed the high-energy diet showed higher triglyceride levels than those in the other two groups. However, at the end of the finisher period, that effect was mitigated and no difference was found among the three experimental diets, confirming the lack of significance detected on carcass fatness. Kim et al. [40], in lighter pigs, did not observe differences between diets with different energy contents. As in the current trial, Suárez-Belloch et al. [29] also found no influence of CP and AA restriction on triglyceride levels. However, in other report, Suárez-Belloch et al. [43] detected that the reduction of CP and AA contents promoted a linear increase of triglycerides at the end of the grower phase.

### 3.3. Serum Sex Hormones

All gilts, both EG and IG, presented basal serum progesterone concentrations (<0.400 ng/mL) when the doses of immunocastration were applied. The day before slaughter, 17% of EG had reached puberty, because these EG showed high levels of progesterone (32.2 and 31.4 ng/mL), while all IG continued to present low levels (<0.600 ng/mL) (data not shown). With the same type of white-breed gilts, Mitjana et al. [48] did not detect differences in this parameter at slaughter between EG and IG, but it has to be noted that the gilts in that study were younger (approximately 125 kg BW). However, with Chinese and Iberian gilts, other authors did find significantly higher concentrations of progesterone in EG than in IG on the preslaughter day [49] or even some months before slaughter [50,51]. The greater effect observed with Chinese and Iberian gilts could be because these breeds reach puberty earlier [52]. Van den Broeke et al. [19] also reported differences in progesterone level in white-breed gilts from just before the second vaccination, but the immunocastration doses were applied later (at 70 and 105 kg BW) than in our trial and in Mitjana’s [48] experiment. Therefore, the gilts of Van den Broeke et al. [19] were more sexually developed when they were immunized against GnRF, and thus the difference between EG and IG was expected to be greater.

Data about serum estradiol concentration are shown in Table 4. No differences (*p* = 0.795) between EG and IG were detected in estradiol levels, corroborating the findings of Van den Broeke et al. [19], Mitjana et al. [48], and Pérez-Ciria et al. [10], and no significant (*p* = 0.787) interaction type of gilt × sampling time was found. Additionally, estradiol concentration increased in serum as gilts grew older (*p* = 0.0005), in agreement with Pérez-Ciria et al. [10].

### 3.4. Reproductive Organs

The IG presented lighter (*p* = 0.015) reproductive tracts than EG, since most organs were lighter (*p* = 0.004 for uterine horns, *p* = 0.010 for uterine corpus, *p* = 0.0001 for cervix, and *p* = 0.024 for vagina) (Table 5). Additionally, ovaries tended to be smaller (*p* = 0.065) and uterine horns (*p* = 0.004), uterine corpus (*p* = 0.022), cervix (*p* = 0.005), and vulva (*p* = 0.021) were shorter in IG than in EG. All of these results indicate that immunization against GnRF inhibited the development of reproductive organs, confirming the results of Hernández-García et al. [50], Dalmau et al. [51], and Mitjana et al. [48].

As can be seen in Table 6, no difference was detected (*p* = 0.324) between EG and IG in the total number of ovarian follicles; however, ovaries of IG showed lower (*p* = 0.034) numbers of big follicles (>6 mm) than those of EG. Additionally, the IG group presented a lower proportion of gilts with intermediate and big follicles than the EG group (*p* = 0.01 and *p* = 0.037, respectively). These results indicate that ovarian activity was less intense in IG, confirming the previous results of Pérez-Ciria et al. [10]. Hernández-García et al. [50] detected greater differences between EG and IG in terms of follicular development, because they did not find any visible follicle in the case of IG, and EG presented 3–11 mm follicles and corpora lutea. On the other hand, Dalmau et al. [51], Xue et al. [49], and Mitjana et al. [48] did find that some IG showed follicles as in the present experiment, although the differences in follicular size between EG and IG were more marked.

Overall, immunocastration was an effective method of preventing puberty, although the effects were more evident in the anatomical development of the reproductive tract than in the sex hormone levels. However, it should be noted that three gilts belonging to the IG group were in the phases of estrus or diestrus at slaughter; these animals were removed from the data analysis and description of serum sex hormones, reproductive organs, and ovarian follicles. The reasons for this are unknown, but it has been also observed in other works. It could be due to the reproductive organs having been taken around 11 weeks after the second dose of immunocastration. Claus et al. [53] observed in male pigs that 10 weeks after the second injection, antibody titers to GnRF were almost as low as before the second dose; this may indicate that some IG could have reverted. As in the current trial, Bohrer et al. [16], who administered the second injection of immunocastration 10 weeks before slaughter, found that some IG reached puberty, while Rodrigues et al. [6], who applied the second dose 6 weeks preslaughter, did not observe that any IG showed signs of estrus. It also has to be considered that since gilts were loose in the pen when the doses of immunocastration were applied, some doses might not have been correctly injected. However, the most plausible explanation is that those gilts did not react to immunocastration doses, and, as Zeng et al. [54] observed, might develop lower GnRF antibody titers than the other IG. The reason for the lower antibody production is not clear.

### 3.5. Carcass Quality

Table 7 shows that no effect (*p* = 0.998) of immunocastration on carcass weight was observed, because the slaughter was at a target BW (close to 135 kg). Carcass yield was also not influenced (*p* = 0.851) by immunization against GnRF, in agreement with previous reports [6,17,19]. However, fat thickness at the GM was greater (*p* = 0.011) in IG, corroborating the findings of a great number of studies in this field [10,17,55]. This result may lead to a decrease in rejected carcasses intended for Teruel dry-cured ham production and optimize the dry-curing process of the pieces [56]. Hams of IG were similar in length (*p* = 0.144) but narrower (*p* = 0.019 for ham perimeter) than those of EG. Immunocastration resulted in a reduction (*p* = 0.034) in the weight of the main pieces, especially the shoulder (*p* = 0.012), but this was not reflected when they were expressed as percentage of carcass (*p* > 0.05). This result agrees with the results of Daza et al. [57], who did not detect differences in these variables. However, Pérez-Ciria et al. [10] observed that IG had lower total yield (ham + shoulder) than EG. The discrepancies in the weights or yields of trimmed cuts between studies may be due to the slaughter criterion (fixed BW or age).

Regarding experimental diets, limited effects on carcass quality were found. The weight and yield of carcasses were not affected (*p* > 0.05) by feeding, probably because of the similar slaughter weight, which confirmed other reports comparing different energy contents or CP and AA levels [7,35,58]. Similarly, diets did not influence (*p* > 0.05) ham size. In terms of carcass fatness, although a thicker fat depth at the GM was expected in animals fed the tested diets, this was not observed (*p* = 0.698). It is worth noting that those animals fed the high-energy diet or the low-CP and -AA diet had 1 mm thicker fat thickness than those fed the control diet, but this result was nonsignificant. Other authors did find differences (Suarez-Belloch et al. [7] increasing in 140 kcal above 2280 kcal/kg; and Sirtori et al. [59] restricting CP and AA contents). The hypothesis being that those nutritional strategies generate an excess of energy/Lys ingested which is then transformed into fat [60,61]. The lack of effect in the present study might have been due to the high variability of data, the shorter experimental time, or the fact that the nutrient levels tested were more prudent. Feeding also had limited effect on the weight of main pieces; only shoulder tended to be lighter (*p* = 0.060) with the diet low in CP and AA than with the other diets. Ruiz-Ascacibar et al. [62] found a similar effect and Suárez-Belloch et al. [29] only observed this effect numerically. No impact (*p* > 0.10) of dietary treatments on ham and shoulder yields was detected.

## 4. Conclusions

Under our experimental conditions, it can be concluded that immunization against GnRF is an interesting strategy to apply in gilts intended for the PDO Teruel ham, because it improves animal growth rate, decreases the number of fattening days, and increases fat thickness at the GM, which is a very desirable aspect for the dry-curing process. On the other hand, in gilts from 76 to 134 kg BW, a rise in dietary energy by 0.15 Mcal of net energy/kg or a drop in dietary CP by 2 percentage points and in AA do not improve carcass fatness, but could be beneficial for pig farmers whether the gilts are entire or immunocastrated; the first dietary strategy improves feed efficiency and the second one does not impair growth performance.

## Figures and Tables

**Table 1:** Estimated and analyzed nutrient composition of the experimental diets (%, as-fed basis).

Item	Grower Diet (76 to 102 kg Body Weight)			Finisher Diet (102 to 134 kg Body Weight)		
	Control	High Energy	Low CP and AA	Control	High Energy	Low CP and AA
Estimated nutrient composition						
Net energy, Mcal/kg	2.33	2.48	2.33	2.33	2.48	2.33
Dry matter	88.3	88.6	88.3	88.4	88.7	88.4
Ash	4.07	4.05	3.89	3.88	3.83	3.68
CP	16.0	16.0	14.0	14.5	14.5	12.5
Ether extract	3.08	6.10	3.00	3.02	5.81	2.81
Neutral detergent fiber	12.3	11.8	13.0	12.9	12.1	13.5
Starch	47.4	45.2	49.9	49.2	47.5	52.0
Digestible AA						
Lysine	0.77	0.77	0.67	0.63	0.63	0.54
Methionine	0.24	0.24	0.21	0.21	0.20	0.18
Methionine + cysteine	0.49	0.49	0.44	0.44	0.43	0.39
Threonine	0.50	0.50	0.43	0.43	0.43	0.36
Tryptophan	0.16	0.16	0.14	0.15	0.15	0.13
Analyzed nutrient composition						
Gross energy, Mcal/kg	3.99	4.12	3.92	3.91	4.12	3.95
Dry matter	88.7	88.2	88.0	88.0	89.4	88.1
Ash	4.18	4.19	4.17	3.85	3.98	3.65
CP	16.2	15.9	14.4	14.5	15.1	12.7
Ether extract	3.55	5.88	3.44	3.00	5.65	3.73
Neutral detergent fiber	10.9	10.2	10.5	10.5	8.96	10.2
Starch	42.1	40.3	44.0	44.5	47.8	49.0
Total AA						
Lysine	0.98	0.98	0.79	0.76	0.77	0.71
Methionine	0.28	0.27	0.25	0.24	0.25	0.23
Threonine	0.62	0.60	0.59	0.56	0.58	0.51

CP: crude protein; AA: amino acids.

**Table 2:** Effect of immunocastration and diet on growth performance (least square means) of heavy gilts.

Item ^1^	Type of Gilt ^2^		SEM ^3^ (*n* = 12)	Diet ^4^			SEM ^3^ (*n* = 8)	*p*-Value ^5^	
	EG	IG		Control	High Energy	Low CP and AA		Gilt	Diet
Body weight, kg									
Initial	40.1	40.5	0.09	40.5	40.2	40.1	0.11	0.004	0.066
First dose	57.0	58.0	0.34	57.7	58.1	56.7	0.41	0.044	0.067
Second dose ^6^	75.3	77.1	0.43	76.2	76.4	75.9	0.53	0.011	0.762
Start finisher period	100.8	103.6	0.74	102.8	102.2	101.7	0.91	0.016	0.680
Day before slaughter	134.0	133.6	1.03	131.9	135.1	134.4	1.26	0.786	0.206
ADG Initial–1st dose, kg/day	0.940	0.974	0.0180	0.954	0.997	0.919	0.0220	0.193	0.068
ADG 1st–2nd dose ^7^, kg/d	0.924 (0.878–0.966)	0.960 (0.917–0.999)	-	0.929 (0.872–0.979)	0.927 (0.870–0.978)	0.970 (0.918–1.016)	-	0.222	0.392
Grower period ^8^									
ADG ^7^, kg/d	0.911 (0.871–0.952)	0.950 (0.909–0.991)	-	0.950 (0.900–1.001)	0.919 (0.870–0.970)	0.921 (0.872–0.972)	-	0.175	0.599
ADFI, kg/d	2.80	2.92	0.051	2.90	2.82	2.86	0.062	0.098	0.635
G:F	0.328	0.324	0.0057	0.328	0.328	0.323	0.0070	0.686	0.846
Finisher period ^9^									
ADG ^7^, kg/d	0.785 (0.754–0.817)	0.872 (0.839–0.906)	-	0.764 ^b^ (0.726–0.802)	0.860 ^a^ (0.820–0.902)	0.861 ^a^ (0.821–0.903)	-	0.001	0.002
ADFI, kg/d	2.90	3.13	0.037	2.99 ^ab^	2.94 ^b^	3.12 ^a^	0.046	0.0005	0.035
G:F	0.271	0.278	0.0059	0.255 ^b^	0.294 ^a^	0.275 ^ab^	0.0072	0.378	0.005
Overall diet period ^10^									
ADG, kg/d	0.837	0.906	0.0138	0.843	0.885	0.886	0.0169	0.002	0.153
ADFI, kg/d	2.86	3.04	0.039	2.95	2.88	3.02	0.047	0.006	0.153
G:F	0.293	0.300	0.0043	0.286 ^b^	0.308 ^a^	0.296 ^ab^	0.0053	0.292	0.037
Overall trial period ^11^									
ADG, kg/d	0.869	0.927	0.0100	0.879	0.910	0.905	0.0123	0.0007	0.185
Length, d	108.6	101.2	1.63	104.6	105.0	105.0	2.00	0.005	0.988

^1^ ADG: average daily gain; ADFI: average daily feed intake; G:F: gain-to-feed ratio. ^2^ EG: entire gilt; IG: immunocastrated gilt. ^3^ SEM: standard error of the mean. ^4^ Grower period: control (2.33 Mcal net energy (NE)/kg, 16% crude protein (CP) and 0.77% standardized ileal digestible (SID) lysine (Lys)); high energy (2.48 Mcal NE/kg, 16% CP and 0.77% SID Lys); and low CP and amino acids (AA) (2.33 Mcal NE/kg, 14% CP and 0.67% SID Lys). Finisher period: control (2.33 Mcal NE/kg, 14.5% CP and 0.63% SID Lys); high energy (2.48 Mcal NE/kg, 14.5% CP and 0.63% SID Lys); and low CP and AA (2.33 Mcal NE/kg, 12.5% CP and 0.54% SID Lys). ^5^ No significant interactions (type of gilt × diet) were found (*p* > 0.05). ^6^ Start of the grower period. ^7^ Statistical evaluation was carried out with data after their transformation. Data are presented as back-transformed least square means with 95% confidence intervals within parentheses. ^8^ From the second dose to approximately 100 kg. ^9^ From approximately 100 kg to the day before slaughter. ^10^ From the second dose to slaughter or when the experimental diets were tested. ^11^ From approximately 40 kg to the day before slaughter. Within a row, means without a common superscript (^a,b^) differ (*p* < 0.05).

**Table 3:** Impact of immunocastration and diet on serum metabolites (least square means) of gilts.

Item	Albumin, g/dL	Urea, mg/dL	Cholesterol, mg/dL
Type of gilt			
Entire	3.34	26.3	73.8
Immunocastrated	3.19	28.7	65.9
SEM ^1^ (*n* = 24)	0.107	1.49	3.04
Diet ^2^			
Control	3.09	30.3	68.6
High energy	3.41	26.0	69.8
Low CP and AA	3.28	26.2	71.1
SEM ^1^ (*n* = 16)	0.131	1.82	3.72
Sampling time			
At the end of the grower period	2.89	25.7	63.2
At the end of the finisher period	3.63	29.3	76.4
SEM ^1^ (*n* = 24)	0.100	1.22	2.87
*p*-value ^3^			
Type of gilt	0.339	0.274	0.087
Diet	0.265	0.187	0.877
Sampling time	0.0001	0.011	0.012

^1^ SEM: standard error of the mean. ^2^ Grower period: control (2.33 Mcal net energy (NE)/kg, 16% crude protein (CP) and 0.77% standardized ileal digestible (SID) lysine (Lys)); high energy (2.48 Mcal NE/kg, 16% CP and 0.77% SID Lys); and low CP and amino acids (AA) (2.33 Mcal NE/kg, 14% CP and 0.67% SID Lys). Finisher period: control (2.33 Mcal NE/kg, 14.5% CP and 0.63% SID Lys); high energy (2.48 Mcal NE/kg, 14.5% CP and 0.63% SID Lys); and low CP and AA (2.33 Mcal NE/kg, 12.5% CP and 0.54% SID Lys). ^3^ No significant interactions (type of gilt × diet, type of gilt × sampling time, diet × sampling time, type of gilt × diet × sampling time) were found (*p* > 0.05).

**Table 4:** Effect of immunocastration on serum estradiol concentration (least square means) of heavy gilts.

Item	Estradiol, pg/mL
Type of gilt	
Entire	27.6
Immunocastrated	26.7
SEM ^1^ (*n* = 36)	2.39
Sampling time	
At first dose of immunocastration	22.0 ^b^
At second dose of immunocastration	27.4 ^ab^
Day before slaughter	32.1 ^a^
SEM ^1^ (*n* = 24)	2.17
*p*-value	
Type of gilt	0.795
Sampling time	0.0005
Type of gilt × sampling time	0.787

^1^ SEM: standard error of the mean. Within a column, means without a common superscript (^a,b^) differ (*p* < 0.05).

**Table 5:** Impact of immunocastration on reproductive organs (least square means) of heavy gilts.

Trait	Type of Gilt		SEM ^1^ (*n* = 12)	*p*-Value
	Entire	Immunocastrated		
Ovaries				
Weight, g	5.85	4.60	0.922	0.354
Size, cm^3^	10.76	7.34	1.213	0.065
Uterine horns ^2^				
Weight, g	106.6 (77.6–140.2)	49.2 (31.1–71.4)	-	0.004
Length, cm	65.6 (57.2–74.7)	47.8 (41.0–55.1)	-	0.004
Uterine corpus ^2^				
Weight, g	5.30 (3.46–7.89)	2.24 (1.29–3.57)	-	0.010
Length, cm	4.10 (3.21–5.22)	2.72 (2.14–3.47)	-	0.022
Cervix				
Weight, g	62.3	29.5	5.03	0.0001
Length, cm	16.0	13.5	0.55	0.005
Vagina				
Weight, g	31.0	21.2	2.87	0.024
Length, cm	10.44	9.38	0.589	0.214
Vestibule length, cm	13.1	12.5	0.31	0.166
Vulva length, cm	3.93	3.15	0.217	0.021
Total reproductive tract weight, g	186.8	117.1	17.80	0.015

^1^ SEM: standard error of the mean. ^2^ Statistical evaluation was carried out with data after their transformation. Data are presented as back-transformed least square means with 95% confidence intervals within parentheses.

**Table 6:** Effect of immunocastration on ovarian follicles of heavy gilts ^1,2^.

Trait	Type of Gilt		*p*-Value
	Entire	Immunocastrated	
Number of follicles			
<2 mm	5.73 (1.25–26.32)	11.60 (2.37–56.82)	0.535
2–4 mm	24.4 (11.9–49.9)	35.8 (16.9–75.6)	0.469
4–6 mm	10.91 (4.68–25.44)	7.50 (3.06–18.39)	0.556
>6 mm	2.00 (0.33–11.94)	0 (0–0)	0.034
Total	43.0 (30.8–60.0)	54.9 (38.8–77.6)	0.324
Gilts with follicles, %			
<2 mm	45.5 (20.3–73.2)	40.0 (15.8–70.3)	0.801
2–4 mm	90.9 (56.1–98.7)	80.0 (45.9–95.0)	0.473
4–6 mm	90.9 (56.1–98.7)	40.0 (15.8–70.3)	0.010
>6 mm	27.3 (9.0–58.6)	0 (0–0)	0.037

^1^ Data are presented as back-transformed least square means with 95% confidence intervals within parentheses. ^2^ *n* = 12.

**Table 7:** Impact of immunocastration and diet on carcass quality (least square means) of heavy gilts.

Trait	Type of Gilt ^1^		SEM ^2^ (*n* = 66)	Diet ^3^			SEM ^2^ (*n* = 44)	*p*-Value ^4^	
	EG	IG		Control	High Energy	Low CP and AA		Gilt	Diet
Slaughter weight, kg	134.3	133.7	1.22	131.9	135.9	134.2	1.49	0.711	0.161
Carcass weight, kg	104.6	104.6	0.93	105.4	103.6	104.9	1.13	0.998	0.509
Carcass yield, %	77.7	77.9	0.69	79.0	76.7	77.8	0.84	0.851	0.137
Fatness at the GM ^5,6^, mm	21.2	23.7	0.66	21.9	22.8	22.6	0.81	0.011	0.698
Ham length ^6^, cm	40.1	39.8	0.14	39.9	40.1	39.9	0.18	0.144	0.531
Ham perimeter ^6^, cm	78.2	77.5	0.22	78.1	77.9	77.5	0.27	0.019	0.299
Trimmed cut weight ^6^, kg									
Ham	13.5	13.2	0.08	13.4	13.5	13.2	0.10	0.087	0.119
Shoulder	8.79	8.63	0.043	8.80	8.71	8.62	0.053	0.012	0.060
Total ^7^	22.2	21.9	0.12	22.2	22.2	21.8	0.14	0.034	0.062
Trimmed cut yield ^6^, % carcass									
Ham	12.9	12.7	0.14	12.8	13.0	12.6	0.17	0.354	0.211
Shoulder	8.48	8.27	0.083	8.38	8.49	8.26	0.102	0.087	0.278
Total ^7^	21.4	21.0	0.22	21.1	21.5	20.8	0.27	0.172	0.187

^1^ EG: entire gilt: IG: immunocastrated gilt. ^2^ SEM: standard error of the mean. ^3^ Grower period: control (2.33 Mcal net energy (NE)/kg, 16% crude protein (CP) and 0.77% standardized ileal digestible (SID) lysine (Lys)); high energy (2.48 Mcal NE/kg, 16% CP and 0.77% SID Lys); and low CP and amino acids (AA) (2.33 Mcal NE/kg, 14% CP and 0.67% SID Lys). Finisher period: control (2.33 Mcal NE/kg, 14.5% CP and 0.63% SID Lys); high energy (2.48 Mcal NE/kg, 14.5% CP and 0.63% SID Lys); and low CP and AA (2.33 Mcal NE/kg, 12.5% CP and 0.54% SID Lys). ^4^ No significant interactions (type of gilt × diet) were found (*p* > 0.05). ^5^ GM: gluteus medius muscle. ^6^ From the left side. ^7^ Ham + shoulder.

## Data Availability

Data available on request due to restrictions of privacy.

## Supplementary Materials

The following are available online at https://www.mdpi.com/article/10.3390/ani11071900/s1, Table S1: Ingredients of the experimental diets (%, as fed-basis).
