# Supplementary material for: Effect of Immunocastration and Diet on Growth Performance, Serum Metabolites and Sex Hormones, Reproductive Organ Development and Carcass Quality of Heavy Gilts
Source: Animals (Basel). 2021 Jun 25;11(7):1900. doi: 10.3390/ani11071900 (PMC8300150; doi:10.3390/ani11071900)
Supplement: Supplementary file 1 [file animals-11-01900-s001.zip › animals-1244334-supplementary.pdf]

animals

MDPI

Article

Effect of Immunocastration and Diet on Growth Performance, Serum Metabolites and Sex Hormones, Reproductive Organs Development and Carcass Quality of Heavy Gilts

Leticia Pérez-Ciria [1], Francisco Javier Miana-Mena [2], María Victoria Falceto [3], Olga Mitjana [3] and Maria Angeles Latorre [1,*]

[1] Departamento de Producción Animal y Ciencia de los Alimentos, Instituto Agroalimentario de Aragón-IA2 (Universidad de Zaragoza-CITA), 50013 Zaragoza, Spain; leticiapcgm@gmail.com (L.P.-C.)
[2] Departamento de Farmacología y Fisiología, Instituto Agroalimentario de Aragón-IA2 (Universidad de Zaragoza-CITA), 50013 Zaragoza, Spain; jmiana@unizar.es (F.J.M.-M.)
[3] Departamento de Patología Animal, Instituto Agroalimentario de Aragón-IA2 (Universidad de Zaragoza-CITA), 50013 Zaragoza, Spain; vfalceto@unizar.es (M.V.F.); omitjana@unizar.es (O.M.)
* Correspondence: malatorr@unizar.es (M.A.L.)

Table S1. Ingredients of the experimental diets [1] (%, as-fed basis).

Ingredient	Grower diet (76 to 102 kg of body weight)			Finisher diet (102 to 134 kg of body weight)		
	Control	High energy	Low CP and AA	Control	High energy	Low CP and AA
Corn	35.0	33.9	35.0	35.0	32.5	35.0
Wheat	18.0	18.0	18.4	17.0	18.0	18.1
Barley	17.6	15.0	21.0	21.1	21.8	25.0
Oat	9.00	8.72	11.0	11.0	8.00	12.0
Soybean meal 47% CP	17.8	18.7	11.9	13.6	14.4	7.69
Palm oil	0.53	3.65	0.34	0.36	3.36	0.08
Calcium carbonate	0.79	0.78	0.80	0.85	0.85	0.86
Sodium chloride	0.45	0.45	0.45	0.45	0.45	0.45
Monocalcium phospate	0.26	0.27	0.31	0.13	0.13	0.18
L-Lysine 50%	0.23	0.21	0.30	0.14	0.12	0.23
L-Threonine	0.02	0.02	0.02	-	-	0.01
DL-Methionine	0.02	0.02	0.01	-	-	-
L-Tryptophan	-	-	-	0.01	0.01	0.01
Premix [2]	0.40	0.40	0.40	0.40	0.40	0.40

[1] Grower diet: control (2.33 Mcal net energy (NE)/kg, 16% crude protein (CP) and 0.77% standardized ileal digestible (SID) lysine (Lys)); high energy (2.48 Mcal NE/kg, 16% CP and 0.77% SID Lys); and low CP and amino acids (AA) (2.33 Mcal NE/kg, 14% CP and 0.67% SID Lys). Finisher diet: control (2.33 Mcal NE/kg, 14.5% CP and 0.63% SID Lys); high energy (2.48 Mcal NE/kg, 14.5% CP and 0.63% SID Lys); and low CP and AA (2.33 Mcal NE/kg, 12.5% CP and 0.54% SID Lys). [2] Provided the following per kilogram of complete diet: 6.5 IU Vitamin A; 1.5 IU Vitamin D3; 15 mg α-tocopherol; 3 mg Vitamin B2; 1 mg Vitamin B6; 0.02 mg Vitamin B12; 15 mg nicotinic acid; 8 mg pantothenic acid; 100 mg choline chloride; 100 mg Zn (ZnO); 50 mg Mn (MnO); 250 mg Fe ($FeCO_3$); 10 mg Cu ($CuSO_4 \cdot 5H_2O$); 0.2 mg Se (Na_2O_3Se); 2 mg BHT; 1 mg I (KI); 500 FYT 6-phytase.
